# Neuromotor frailty phenotype and fall risk in geriatrics with knee osteoarthritis: a cross-sectional analysis of physical therapy performance and psychometric outcomes

**DOI:** 10.3389/fmed.2026.1772639

**Published:** 2026-03-23

**Authors:** Batool Abdulelah Alkhamis, Basant Hamdy Elrefaey, Ravi Shankar Reddy, Wafaa Mahmoud Amin, Esraa M. Fayed, Joud Fahad Albishri, Taif Ibrahim Asiri, Abdulaziz Ali Matiq, Hany Mahmoud Fares

**Affiliations:** 1 Program of Physical Therapy, Department of Medical Rehabilitation Sciences, College of Applied Medical Sciences, King Khalid University, Abha, Saudi Arabia; 2 Department of Physical Therapy for Cardiovascular/Respiratory Disorder and Geriatrics, Faculty of Physical Therapy, Cairo University, Giza, Egypt; 3 Department of Physical Therapy, College of Nursing and Health Sciences, Jazan University, Jazan, Saudi Arabia; 4 Department of Basic Science for Physical Therapy, Faculty of Physical Therapy, Cairo University, Giza, Egypt; 5 Department of Physical Therapy for Paediatrics, Faculty of Physical Therapy, Cairo University, Giza, Egypt; 6 Department of Physical Therapy, Abha International Private Hospital, Abha, Saudi Arabia

**Keywords:** falls, geriatric assessment, knee osteoarthritis, mobility limitation, physical therapy modalities, postural balance

## Abstract

**Background:**

Falls are a leading cause of morbidity in older adults with knee osteoarthritis (KOA), yet current assessment strategies often lack the specificity needed to identify those at the most significant risk. The integration of physical therapy (PT) outcome measures may enhance fall risk stratification by capturing functional impairments across multiple domains. To compare neuromotor frailty profiles between older adult fallers and non-fallers with KOA using standardized PT assessments, and to evaluate the predictive validity and latent structure of these measures for fall risk classification.

**Methods:**

In this cross-sectional study, 128 community-dwelling older adults with KOA (64 fallers, 64 non-fallers) completed a battery of PT outcome measures, including the Timed Up and Go Dual Task (TUG-DT), Four Square Step Test (FSST), Falls Efficacy Scale–International (FES-I), Functional Gait Assessment (FGA), Short Physical Performance Battery (SPPB), and others. Group differences were assessed using independent *t*-tests, logistic regression was employed to examine variables independently associated with fall status, and exploratory factor analysis (EFA) was conducted to examine the underlying constructs.

**Results:**

Fallers showed significantly poorer performance across all PT measures, including slower TUG-DT (17.90 ± 3.42 vs. 13.42 ± 3.10 s, *p* < 0.001) and lower FGA scores (16.45 ± 4.16 vs. 20.71 ± 3.87, *p* < 0.001). TUG-DT (OR = 1.35, 95% CI: 1.18–1.55), FSST (OR = 1.21, CI: 1.05–1.39), and FES-I (OR = 1.11, CI: 1.03–1.19) were independently associated with fall status. EFA identified three distinct factors: dynamic balance, functional mobility, and fear of falling.

**Conclusion:**

A multidimensional battery of PT outcome measures effectively differentiates fallers from non-fallers and characterizes the neuromotor frailty phenotype in older adults with KOA. These measures demonstrate strong discriminative performance for fall status and provide clinically relevant indicators of fall risk as a multidimensional construct.

## 1 Introduction

Falls remain one of the leading causes of injury, decline in function, and loss of independence among older adults, with severe impacts on illness, death, and health care costs (1). In individuals with musculoskeletal conditions, particularly knee osteoarthritis (KOA), the risk of falling is significantly increased by joint pain, limited mobility, weaker lower-limb muscles, and impaired balance control (2). KOA affects approximately 10%–15% of adults over 60 and is a major contributor to activity limitations and disability in older populations (3). In clinical practice, physical therapists often lead the management of functional problems associated with KOA, with an increasing emphasis on fall prevention as a key goal (4–8). Although falls are common and have serious consequences in this group, fall risk screening tools are typically general and may overlook specific neuromotor issues in older adults with KOA (9). This highlights the need for targeted, population-specific assessment methods that integrate performance tests and self-reports to thoroughly evaluate functional risks.

Previous studies have shown that older adults with KOA walk more slowly, exhibit poorer balance, and experience lower-limb muscle weakness, all of which independently increase the risk of falls (9–11). Tests such as the Timed Up and Go (TUG), Four Square Step Test (FSST), and Short Physical Performance Battery (SPPB) are well-validated in geriatric and musculoskeletal populations as reliable measures of functional mobility and fall risk (10–12). In addition, dual-task assessments, including the TUG with an added cognitive or motor task (TUG-DT), have demonstrated better sensitivity in detecting individuals with impaired neuromotor control, which is especially relevant in real-world settings where divided attention is common (13). Psychological factors, such as fear of falling, as assessed with instruments such as the Falls Efficacy Scale–International (FES-I), are also independently associated with falls and functional decline (14, 15). However, many studies have examined these physical and psychological factors separately, with limited integration into comprehensive assessment models tailored for older adults with KOA (7, 16–20).

Given the complex interaction of biomechanical, neuromuscular, and perceptual factors contributing to falls in this population, there is a vital need to examine how standardized physical therapy outcome measures relate to fall risk and how they may cluster into underlying dimensions of neuromotor frailty (21). While existing fall risk models often include isolated variables, few studies have employed an integrated analytical approach that captures the multifactorial nature of frailty and functional decline in KOA (22). Moreover, there is limited evidence on the relative diagnostic accuracy of individual PT assessments or on the extent to which their combined use improves fall-risk stratification in this high-risk group (23). Addressing this gap has practical value in enhancing early detection and guiding rehabilitation planning tailored to the functional deficits most predictive of falls. Beyond addressing a methodological gap, the present study contributes novel insight by integrating performance-based mobility tests, dual-task paradigms, and psychometric measures within a unified analytical framework to characterize a neuromotor frailty phenotype specific to older adults with knee osteoarthritis. While previous studies have examined individual fall-related variables in isolation, few have combined multivariate modeling, discriminative accuracy analysis, and exploratory factor analysis to delineate underlying functional domains within this population. This comprehensive approach enhances conceptual clarity and provides clinically actionable evidence for rehabilitation-focused fall risk stratification.

The objective of this study was 2-fold: first, to compare the physical performance and psychological profiles of fallers and non-fallers with KOA using a standardized set of physical therapy outcome measures; and second, to evaluate the predictive utility and underlying structure of these measures in identifying fall risk. In the present study, the term “neuromotor frailty phenotype” is conceptually grounded in established physical frailty models, particularly the Fried frailty phenotype, which characterizes frailty through measurable deficits in physical performance domains such as weakness, slowness, and reduced activity. However, rather than applying the traditional criteria of weight loss, exhaustion, grip strength, gait speed, and activity alone, we operationalized frailty within a neuromotor and rehabilitation-specific framework tailored to KOA. This framework integrates objective measures of dynamic balance, dual-task mobility, lower limb strength, gait performance, and perceived fear of falling to reflect impairments in neuromuscular control and functional stability. Thus, the neuromotor frailty phenotype described in this study represents a domain-specific extension of the physical frailty construct, emphasizing performance-based and psychometric markers most relevant to fall vulnerability in older adults with musculoskeletal pathology. It was hypothesized that fallers would show significantly poorer performance across all functional areas, and that exploratory factor analysis would identify distinct, clinically meaningful constructs—such as dynamic balance, functional mobility, and fear of falling—that characterize neuromotor frailty in this population.

## 2 Materials and methods

### 2.1 Study setting, ethics, and study design

This cross-sectional study was conducted between January 2024 and December 2024 at the Musculoskeletal Rehabilitation Clinic, Department of Physical Therapy, King Khalid University, Kingdom of Saudi Arabia. Ethical approval was obtained from the Institutional Review Board of King Khalid University (ECM#2023-3209), and written informed consent was obtained from all participants before enrollment. All procedures adhered strictly to the ethical standards outlined in the Declaration of Helsinki.

### 2.2 Participants

Participants were recruited through consecutive sampling, with eligible individuals either referred by attending clinicians or identified during scheduled outpatient consultations between January and June 2024. Recruitment and baseline assessments were completed within this period, while data verification, statistical analysis, and manuscript preparation continued through December 2024. All participants were community-dwelling older adults with a confirmed diagnosis of KOA, based on the clinical and radiographic criteria of the American College of Rheumatology (ACR) (24), including persistent knee pain, crepitus on active motion, morning stiffness under 30 min, and radiographic evidence of joint space narrowing or osteophyte formation (Kellgren-Lawrence grade ≥ 2) (25).

Inclusion criteria required participants to be aged 65–85 years, have unilateral or bilateral KOA, and demonstrate independent ambulation, with or without an assistive device, for at least 10 meters. Participants were also required to be cognitively intact, as determined by a score of 24 or higher on the Mini-Mental State Examination (MMSE), to ensure reliable self-reporting and comprehension of assessment tasks. Individuals were excluded if they had a history of total knee arthroplasty, inflammatory arthritic conditions (e.g., rheumatoid arthritis), neurological or vestibular disorders affecting balance, recent lower limb fractures or surgeries (<6 months), or uncontrolled systemic illnesses that could compromise safe participation in performance-based testing. Initial eligibility was confirmed through a standardized screening protocol that included a review of medical history, a clinical examination, and verification of radiographic reports. All participants underwent baseline assessments before data collection to ensure they met the inclusion criteria and were physically able to complete the study’s outcome measures without incurring adverse risks.

### 2.3 Fall status

The primary outcome variable in this study was fall status, defined as the occurrence of one or more unintentional falls in the previous 12 months. In this study, “fall status” refers specifically to the retrospective classification of participants as fallers or non-fallers based on reported fall occurrence within the past 12 months. In contrast, “fall risk” is used conceptually to describe the broader construct of functional vulnerability inferred from physical performance and psychometric measures. Regression and ROC analyses, therefore, examine associations with fall status, while fall risk is interpreted as a multidimensional clinical characteristic rather than a directly observed outcome. A fall was operationally defined in accordance with the World Health Organization’s definition (26): “an event which results in a person coming to rest inadvertently on the ground, floor, or other lower level, not as a result of a major intrinsic event (such as a stroke) or overwhelming hazard.” Fall status was assessed using a structured, interviewer-administered fall history questionnaire, conducted face-to-face by trained physiotherapists. The questionnaire included specific prompts regarding the number of falls, timing, location, circumstances (during ambulation, transferring, or turning), and any resulting injuries, modeled on validated tools used in previous cohort studies (27, 28). To reduce recall bias, participants were guided through a calendar landmarking technique that encouraged the retrieval of events by referencing public holidays, family gatherings, and routine healthcare visits. Participants were subsequently classified into two mutually exclusive groups: fallers (those who reported one or more falls within the past 12 months) and non-fallers (those who reported no falls during the same period). Although some clinical literature distinguishes between single fallers and recurrent fallers (≥2 falls), the present study employed a dichotomous classification (≥1 fall vs. no falls) to maintain consistency with epidemiological conventions and to preserve adequate statistical power for multivariate and factor-analytic procedures. The number of recurrent fallers within the sample was insufficient to permit stable subgroup analyses without risking model overfitting. Future studies with larger samples should examine whether recurrent fallers exhibit distinct neuromotor or psychological profiles compared with single fallers in older adults with knee osteoarthritis. This dichotomization is consistent with epidemiological conventions and has demonstrated predictive validity in previous studies examining functional and psychological correlates of fall risk in older adults with chronic joint conditions (29, 30).

### 2.4 Timed up and go – dual task (TUG-DT)

The TUG-DT was used to evaluate functional mobility under cognitive load, a recognized risk factor for falls among older adults with musculoskeletal impairments (31, 32). The cognitive task consisted of reciting alternating letters of the alphabet (e.g., A, C, E…), a standardized dual-task paradigm commonly used in geriatric mobility research to impose executive and attentional demands during gait (31, 32). Alternating letter generation requires updating working memory, cognitive flexibility, and inhibitory control, thereby producing measurable cognitive–motor interference relevant to real-world ambulation. This approach has been widely adopted in dual-task TUG protocols and has demonstrated construct validity and sensitivity in detecting mobility impairments and fall vulnerability among older adults (31, 32). Participants were instructed to perform the standard Timed Up and Go (TUG) test—which involves standing up from a chair, walking 3 m, turning around, returning, and sitting down—while concurrently reciting the letters of the alphabet in alternating order (e.g., A, C, E). Timing began when the participant initiated movement from the chair and ended upon returning to a seated position. The TUG-DT score was recorded in seconds, with longer completion times reflecting reduced dual-task mobility.

### 2.5 Four Square Step Test (FSST)

The FSST was administered to assess dynamic balance, coordination, and the ability to change direction while stepping (33). Participants were instructed to step as quickly as possible through four squares arranged in a cross configuration using low, flat barriers (canes) placed on the floor. The stepping sequence followed a predetermined order (forward, right, backward, and left), and timing began once the first foot entered square 2 and ended upon exiting square 1. Two trials were conducted, and the fastest time (in seconds) was recorded. Errors such as touching the canes or stepping out of sequence resulted in test repetition (33).

### 2.6 10 -Meter Walk Test (gait speed)

Gait speed was measured using the 10-Meter Walk Test (10MWT), which is widely regarded as a critical indicator of functional capacity and a predictor of fall risk and mobility-related disability in geriatric and orthopedic populations (34). Participants walked at their usual pace along a 10-m path, with the central 6 m timed to avoid acceleration and deceleration. Timing was performed using a digital stopwatch, and the average gait speed (in meters per second) was calculated from two trials. A slower gait speed has been consistently associated with increased frailty, higher fall risk, and poorer physical performance in older adults with lower-extremity osteoarthritis (35).

### 2.7 30 -s chair stand test

The 30-s chair stand test was used to assess lower-limb muscle strength and endurance (36). Participants were seated in a standard-height chair (approximately 43–45 cm) with their arms crossed over their chest. They were instructed to rise to a full standing position and return to a sitting position as many times as possible within 30 s. The total number of completed stands was recorded, with higher counts reflecting greater lower extremity function. This test has been shown to have excellent inter-rater reliability and criterion validity for assessing lower-limb strength in older adults and in those with functional limitations due to KOA (36).

### 2.8 Functional Gait Assessment (FGA)

The FGA was administered to evaluate postural stability and dynamic balance during gait across multiple tasks of increasing complexity (37). The assessment consists of 10 items, including walking with head turns, gait with a narrow base of support, and stepping over obstacles. Each item is scored from 0 to 3, with a maximum score of 30; lower scores indicate poorer performance and greater gait instability. The FGA has demonstrated high sensitivity and specificity for fall prediction among older adults with balance disorders and lower limb musculoskeletal conditions (38).

### 2.9 Short Physical Performance Battery (SPPB)

The SPPB was used as a composite measure of lower extremity function. It includes three subtests: a balance test (side-by-side, semi-tandem, and tandem stands), a gait speed test (4-m walk), and a chair stand test (five consecutive rises) (39). Each subtest is scored from 0 to 4, resulting in a total score of 12. Higher scores indicate better physical function. The SPPB has been extensively validated in geriatric and rehabilitation populations and is a well-established predictor of disability, institutionalization, and falls (40). Standardized protocols from the National Institute on Aging were followed for administration and scoring (40).

### 2.10 Falls Efficacy Scale–International (FES-I)

Fear of falling was assessed using the Falls Efficacy Scale–International (FES-I), a widely validated self-report instrument developed by the Prevention of Falls Network Europe (ProFaNE) (15). The FES-I comprises 16 items assessing participants’ concern about falling during various daily activities, such as cleaning, walking on uneven surfaces, and social interactions. Each item is rated on a four-point Likert scale from 1 (“not at all concerned”) to 4 (“very concerned”), yielding a total score of 16–64. Trained assessors administered the questionnaire face-to-face to minimize misunderstandings and accommodate any literacy or visual limitations. Standardized administration protocols recommended by ProFaNE were followed to ensure inter-individual comparability. The FES-I was used both as an independent variable in multivariate models and as a continuous outcome reflecting psychological frailty (15).

### 2.11 Western Ontario and McMaster Universities Osteoarthritis Index (WOMAC)

The WOMAC was used to evaluate self-reported pain, stiffness, and functional impairment specifically related to KOA (41). This disease-specific instrument comprises 24 items, distributed across three subscales: Pain (5 items), Stiffness (2 items), and Physical Function (17 items). Each item is rated on a five-point Likert scale ranging from 0 (“none”) to 4 (“extreme”), and the item scores are summed to generate subscale totals. The total WOMAC score (range: 0–96) was calculated by summing all subscale scores, with higher values indicating more severe osteoarthritis-related symptoms and disability (41). The WOMAC has been extensively validated in patients with KOA and is recognized as a core outcome measure by the Osteoarthritis Research Society International (OARSI) (42). The questionnaire was administered in Arabic using a linguistically and culturally validated version to ensure comprehension and reliability among participants. Total WOMAC scores were used as a secondary descriptive variable to characterize the clinical severity of KOA within the faller and non-faller groups (42).

### 2.12 Covariates and clinical characteristics

Demographic and clinical variables considered as covariates included age, sex, body mass index (BMI), number of comorbidities, and physical activity level. BMI was calculated as weight (in kilograms) divided by height (in meters) squared. Comorbidities were recorded via self-report and confirmed in medical records. Physical activity was quantified using the International Physical Activity Questionnaire – Short Form (IPAQ-SF), which estimates weekly energy expenditure in MET-minutes. Trained physical therapists administered all assessments in accordance with standardized protocols to ensure consistency and data integrity. Prior to data collection, all assessors underwent structured training sessions led by the principal investigator to standardize test administration, scoring criteria, and safety procedures. A detailed operations manual outlining step-by-step instruction for each outcome measure was used to ensure procedural consistency. To enhance reliability, pilot assessments were conducted prior to participant enrollment, during which inter-rater agreement was evaluated for performance-based measures. Agreement exceeded 0.85 (intraclass correlation coefficient) across key mobility and balance outcomes, indicating high inter-rater reliability. Throughout the study period, periodic monitoring and case discussions were conducted to minimize procedural drift.

### 2.13 Sample size calculation

The sample size was calculated *a priori* using G*Power 3.1.9.7 for a two-tailed independent samples *t*-test comparing fallers and non-fallers on gait speed, assuming a moderate effect size (Cohen’s *d* = 0.50), α = 0.05, and power = 0.80. A minimum of 128 participants (64 per group) was required, with the target increased to 142 to account for potential attrition. This sample was sufficient to support all planned analyses, including between-group comparisons and ROC curve evaluations. In addition to supporting between-group comparisons, the final sample size (*n* = 128) was adequate for multivariate logistic regression and exploratory factor analysis. For logistic regression, the number of fall events (*n* = 64) met the recommended minimum of 10 events per predictor variable in the primary model, thereby reducing the risk of model overfitting. For exploratory factor analysis, the participant-to-variable ratio exceeded 10:1, based on the number of physical therapy outcome measures included in the model, meeting established methodological recommendations for stable factor extraction.

### 2.14 Data analysis

All statistical analyses were performed using SPSS software version 24.0 (IBM Corp., Armonk, NY, United States). Descriptive statistics were used to summarize participant characteristics and physical therapy outcome measures, with results presented as means and standard deviations for continuous variables. Independent-samples *t*-tests were conducted to compare fallers and non-fallers on demographic, clinical, and performance-based variables. Pearson correlation coefficients were calculated to examine the strength and direction of associations among key physical therapy outcomes. A multivariate binary logistic regression model was used to identify independent predictors of fall status, with odds ratios and 95% confidence intervals reported for each significant variable. The WOMAC total score was excluded from the primary regression model because it reflects overall osteoarthritis symptom severity (pain, stiffness, and self-reported functional limitation) rather than objective neuromotor performance. The regression analysis was designed to examine the independent associations of performance-based and fall-related psychometric measures with fall status. Including WOMAC alongside these measures was considered conceptually redundant and potentially collinear with physical performance outcomes; therefore, it was retained as a descriptive clinical variable rather than a predictive covariate. Prior to model estimation, multicollinearity diagnostics were conducted among all independent variables. Variance inflation factors (VIFs) and tolerance statistics were calculated, with VIFs < 5 and tolerances > 0.20 considered acceptable. All predictors met these criteria, indicating no evidence of problematic multicollinearity. To account for potential confounding, a second adjusted multivariate logistic regression model was constructed, including age, sex, body mass index, Kellgren–Lawrence radiographic grade, number of comorbidities, and physical activity level (IPAQ-SF) as covariates. These variables were selected *a priori* based on significant between-group differences and their established associations with fall risk in older adults with KOA. Receiver Operating Characteristic (ROC) curve analysis was performed to assess the discriminative ability of key measures, with the area under the curve (AUC), sensitivity, specificity, and optimal cutoff points calculated using the Youden Index. Additionally, exploratory factor analysis was conducted using principal component analysis with Varimax rotation to identify latent constructs underlying the neuromotor frailty phenotype. All statistical tests were two-tailed, and a *p*-value of less than 0.05 was considered statistically significant. The suitability of the dataset for factor analysis was evaluated using the Kaiser–Meyer–Olkin (KMO) measure of sampling adequacy and Bartlett’s test of sphericity prior to extraction to ensure that the correlation matrix was appropriate for dimensionality reduction. The data used for this study are provided in the Supplementary Table 1.

## 3 Results

Participants with a recent history of falls demonstrated significantly greater age, higher body mass index, and a greater proportion of advanced radiographic KOA (Kellgren-Lawrence grade III–IV) compared to non-fallers (Table 1). Clinically meaningful differences were also observed in comorbidity burden and polypharmacy, with fallers presenting with more comorbid conditions (mean ± SD: 2.18 ± 1.01 vs. 1.67 ± 0.88; *p* = 0.001) and a higher prevalence of taking five or more medications (54.69% vs. 32.81%; *p* = 0.016). Additionally, fallers reported significantly lower levels of physical activity (mean MET-min/week: 1020.45 ± 345.18 vs. 1213.67 ± 389.26; *p* = 0.029) and higher fear of falling, as indicated by elevated FES-I scores (32.45 ± 5.89 vs. 26.71 ± 4.94; *p* < 0.001). No statistically significant differences were observed in sex distribution, use of assistive devices, or use of pain medication. These findings collectively suggest that advanced age, obesity, joint degeneration, medical complexity, and fear of falling are distinguishing clinical features associated with fall status in this population (Table 1).

**TABLE 1:** Demographic and clinical characteristics of participants.

Variable	Fallers (*n* = 64)	Non-fallers (*n* = 64)	*P*-value
Age (years)	74.21 ± 5.34	71.13 ± 4.76	0.002
Sex (female)	44 (68.75%)	39 (60.94%)	0.371
Body mass index (kg/m^2^)	29.83 ± 4.12	27.56 ± 3.77	0.005
Kellgren-Lawrence grade (III–IV)	40 (62.50%)	28 (43.75%)	0.048
Number of comorbidities	2.18 ± 1.01	1.67 ± 0.88	0.001
Polypharmacy (≥5 medications)	35 (54.69%)	21 (32.81%)	0.016
Fall history (past 12 months)	64 (100%)	0 (0%)	< 0.001
IPAQ physical activity level (MET-min/week)	1020.45 ± 345.18	1213.67 ± 389.26	0.029
Assistive device use	18 (28.13%)	11 (17.19%)	0.127
Pain medication use	46 (71.88%)	38 (59.38%)	0.218
Fear of falling (FES-I score)	32.45 ± 5.89	26.71 ± 4.94	0.000

BMI, body mass index; FES-I, Falls Efficacy Scale–International; IPAQ, International Physical Activity Questionnaire; MET, metabolic equivalent of task.

Fallers demonstrated significantly poorer performance across all physical therapy outcome measures compared to non-fallers, highlighting marked deficits in lower limb strength, dynamic balance, gait speed, and functional mobility (Table 2). Specifically, fallers exhibited slower times on the FSST, TUG, and TUG-DT, as well as reduced gait velocity and fewer repetitions on the 30-s chair stand test, all with *p* ≤ 0.003, indicating substantial impairments in neuromotor and dual-task capacities. Composite measures of function, such as the SPPB and FGA, were notably lower among fallers, suggesting broader mobility limitations. Furthermore, elevated FES-I scores and higher WOMAC total scores in the faller group reflect greater fear of falling and worse self-reported pain and functional difficulty, respectively. Although WOMAC scores differed significantly between groups, this variable was intentionally excluded from the multivariate regression model to maintain focus on neuromotor and functional performance constructs rather than overall disease severity. This approach enabled clearer interpretation of the associations between objective mobility impairments and fall status without over-adjusting for the inherent symptom burden of KOA.

**TABLE 2:** Group comparisons of physical therapy outcome measures between fallers and non-fallers.

Outcome measure	Fallers (*n* = 64)	Non-fallers (*n* = 64)	*P*-value
30-s chair stand test (reps)	10.38 ± 2.71	12.75 ± 2.93	0.001
Four Square Step Test (s)	15.62 ± 4.85	12.08 ± 3.91	0.003
10-Meter Walk Test (m/s)	0.83 ± 0.18	1.07 ± 0.21	<0.001
Timed Up and Go (s)	13.48 ± 2.95	10.87 ± 2.46	<0.001
Timed Up and Go – Dual Task (s)	17.90 ± 3.42	13.42 ± 3.10	<0.001
Functional Gait Assessment (score/30)	16.45 ± 4.16	20.71 ± 3.87	<0.001
Short Physical Performance Battery (score/12)	6.83 ± 1.42	8.75 ± 1.64	<0.001
Falls Efficacy Scale–International (score/64)	32.45 ± 5.89	26.71 ± 4.94	<0.001
WOMAC – total score (score/96)	59.37 ± 10.34	46.23 ± 9.78	<0.001

FSST, Four Square Step Test; FGA, Functional Gait Assessment; SPPB; Short Physical Performance Battery; FES-I, Falls Efficacy Scale–International; TUG, Timed Up and Go; TUG-DT, Timed Up and Go – Dual Task; WOMAC, Western Ontario and McMaster Universities Osteoarthritis Index.

**FIGURE 1:**
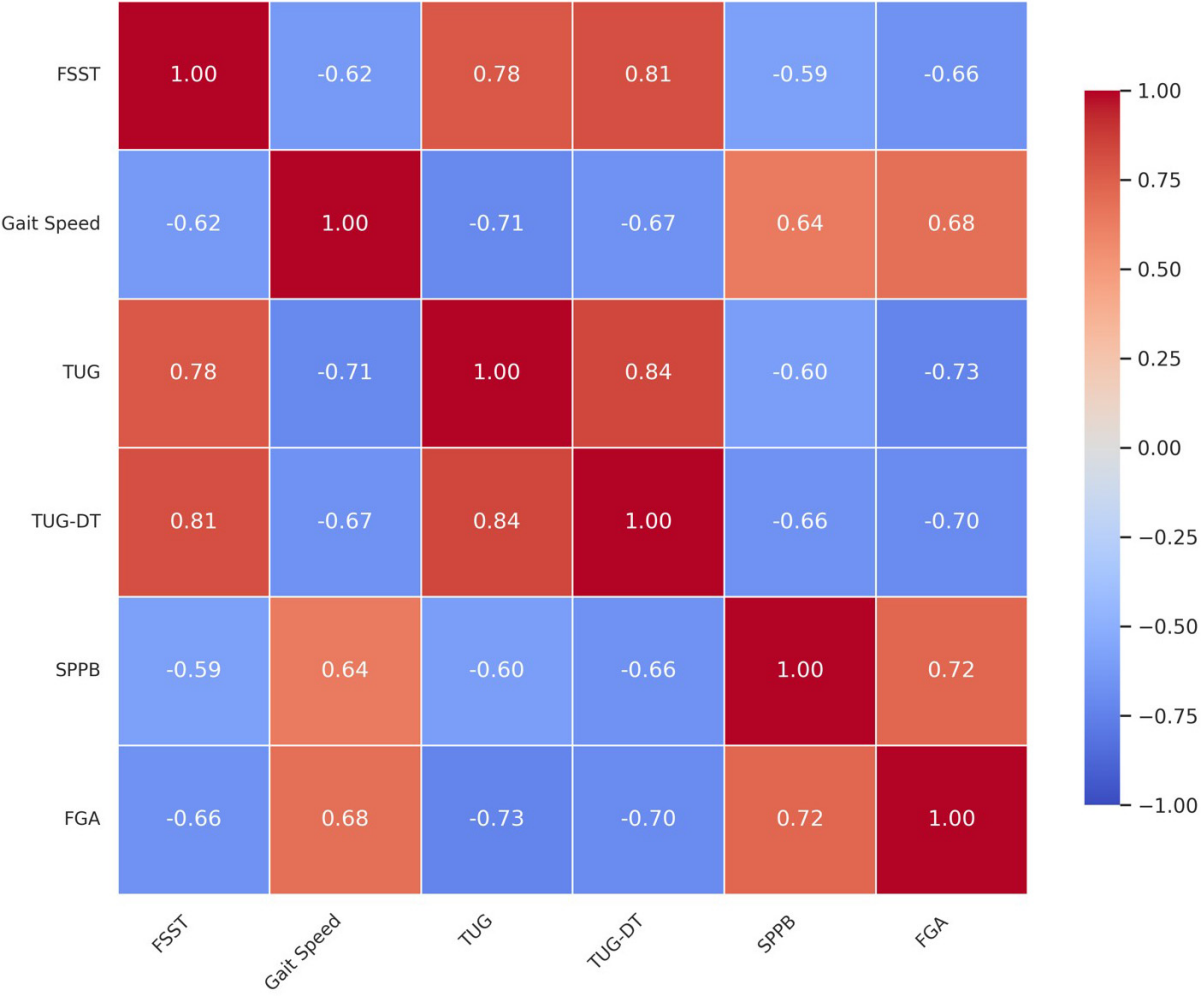
Pearson correlation matrix of physical therapy outcome measures highlighting interrelationships among mobility, balance, and functional tests in older adults with knee osteoarthritis.

Strong intercorrelations were found among physical therapy outcome measures, particularly between TUG and TUG-DT (*r* = 0.84) and between FSST and TUG-DT (*r* = 0.81), indicating high convergence among mobility and dual-task performance tests (Figure 1). Gait speed was moderately to strongly correlated with FGA (*r* = 0.68) and inversely correlated with FSST (*r* = −0.62) and TUG (*r* = −0.71), supporting its utility as a global indicator of functional capacity. The SPPB showed moderate positive correlations with both FGA (*r* = 0.72) and gait speed (*r* = 0.64), and an inverse correlation with TUG-DT (*r* = −0.66) and FSST (*r* = −0.59).

**FIGURE 2:**
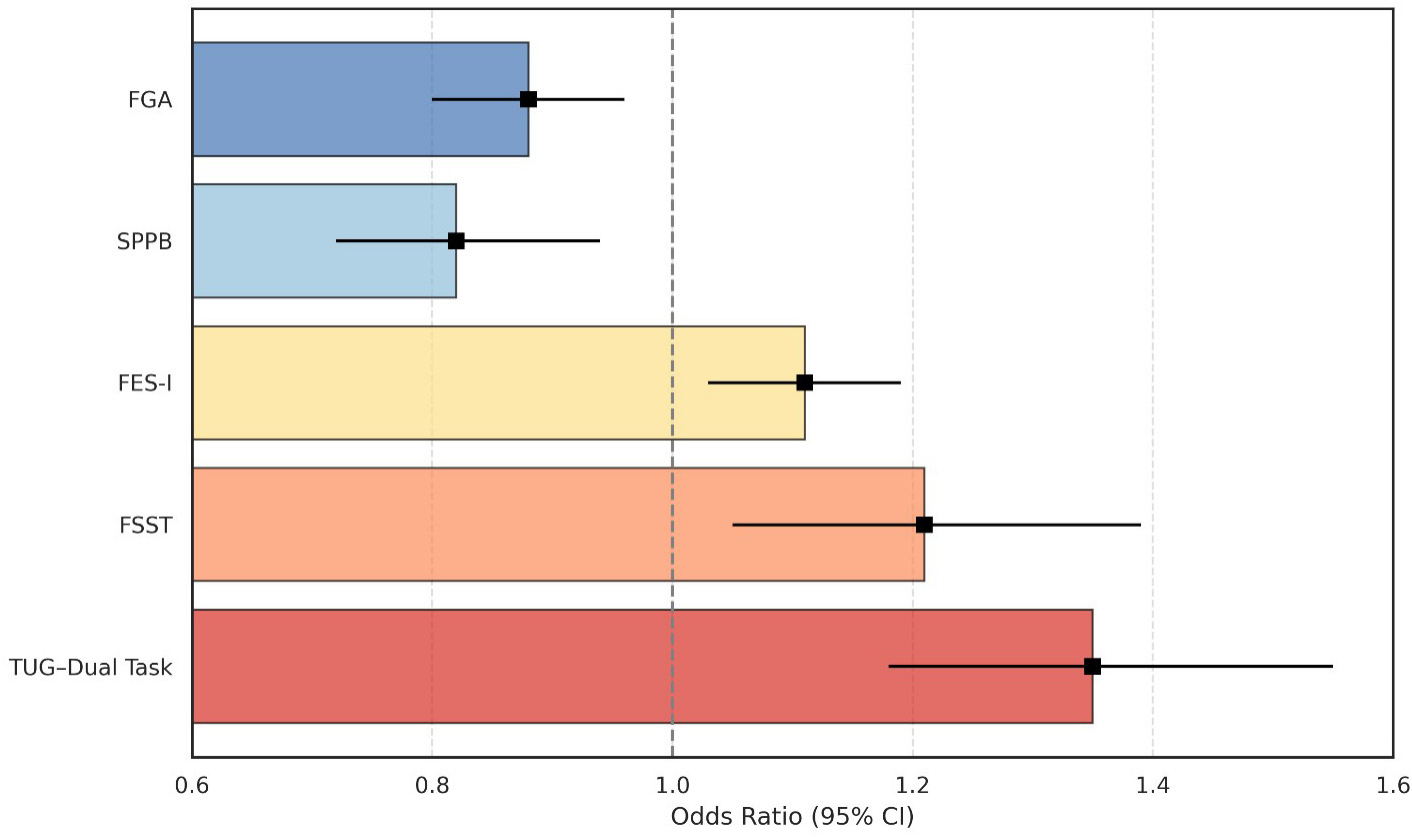
Multivariate logistic regression predicting fall status based on key physical therapy outcome measures in older adults with knee osteoarthritis.

Multivariate logistic regression identified several physical therapy outcome measures as independently associated with fall status, with the model demonstrating a good overall fit (Nagelkerke R^2^ = 0.47) (Figure 2 and Table 3). Multicollinearity diagnostics confirmed acceptable independence among predictors (all VIF values < 3.0), despite strong bivariate correlations between certain mobility measures, supporting the stability of the regression coefficients. After adjustment for age, sex, body mass index, Kellgren–Lawrence grade, comorbidity burden, and physical activity level, the associations between TUG-DT, FSST, FES-I, SPPB, and FGA and fall status remained statistically significant, with only minor attenuation of effect sizes. These findings indicate that the observed associations were not solely attributable to demographic or clinical differences between groups. The TUG-DT showed the strongest association with fall status, with each additional second increasing the odds of being classified as a faller by 35% (OR = 1.35, 95% CI: 1.18–1.55, *p* < 0.001). Similarly, the FSST (OR = 1.21, 95% CI: 1.05–1.39, *p* = 0.008) and FES-I (OR = 1.11, 95% CI: 1.03–1.19, *p* = 0.007) were positively associated with fall status, reflecting impaired dynamic balance and greater fear of falling, respectively. In contrast, higher scores on the SPPB (OR = 0.82, 95% CI: 0.72–0.94, *p* = 0.005) and FGA (OR = 0.88, 95% CI: 0.80–0.96, *p* = 0.002) were protective, indicating that better physical performance and gait stability significantly reduced the likelihood of falls (Table 3).

**TABLE 3:** Multivariate logistic regression predicting fall status using physical therapy outcome measures (fallers vs. non-fallers as dependent variable).

Predictor variable	Odds ratio (OR)	95% confidence interval (CI)	*P*-value
Four Square Step Test (FSST, per second)	1.21	1.05–1.39	0.008
TUG – Dual Task (TUG-DT, per second)	1.35	1.18–1.55	<0.001
Falls Efficacy Scale–International (FES-I, per point)	1.11	1.03–1.19	0.007
Short Physical Performance Battery (SPPB, per point)	0.82	0.72–0.94	0.005
Functional Gait Assessment (FGA, per point)	0.88	0.80–0.96	0.002

Model fit, Nagelkerke R^2^ = 0.47. FSST, Four Square Step Test; FGA, Functional Gait Assessment; SPPB, Short Physical Performance Battery; FES-I, Falls Efficacy Scale–International; TUG-DT, Timed Up and Go – Dual Task; OR, odds ratio; CI, confidence interval.

**FIGURE 3:**
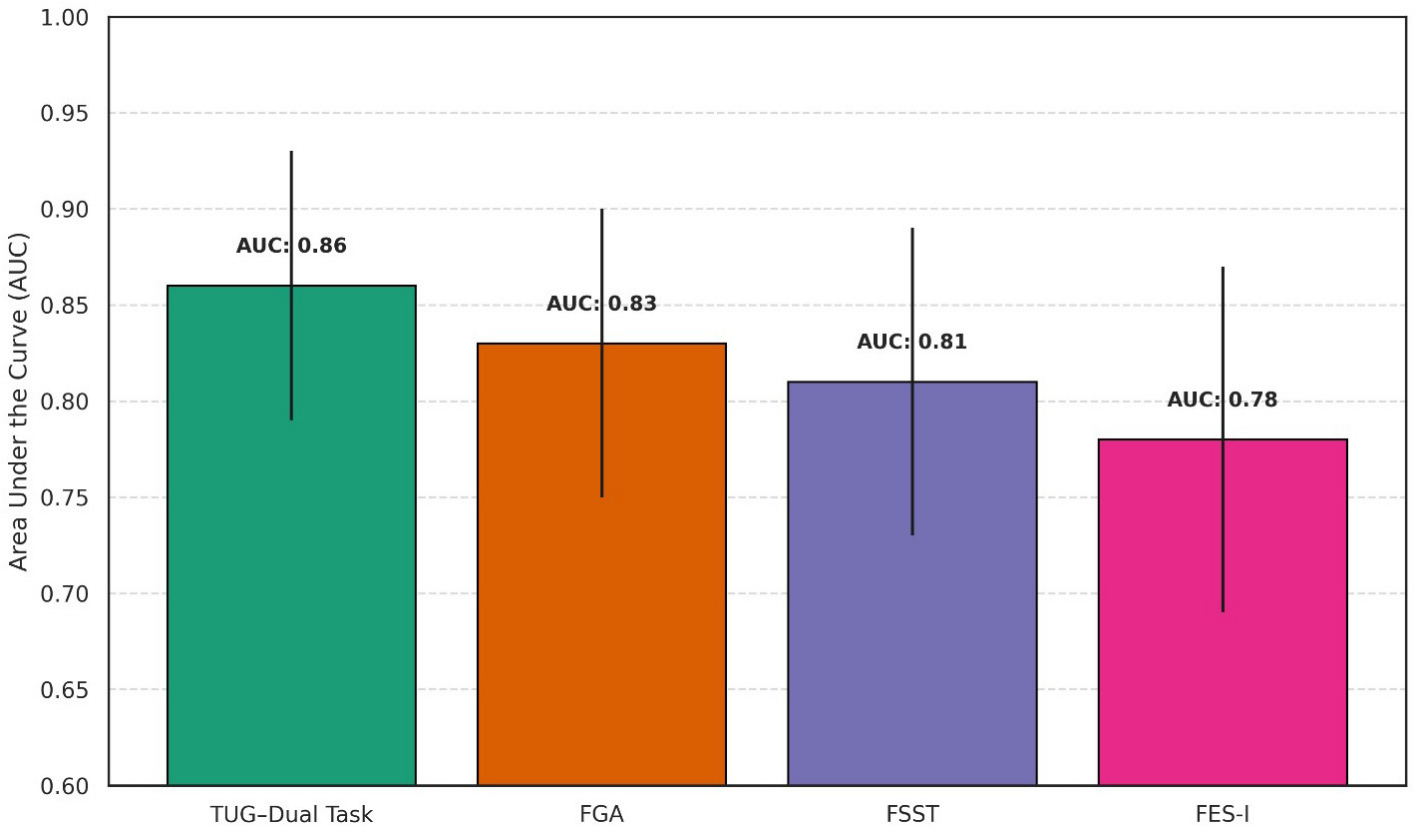
Area under the curve (AUC) comparisons from Receiver Operating Characteristic (ROC) analysis of key physical therapy outcome measures for identifying fallers among older adults with knee osteoarthritis.

Receiver operating characteristic (ROC) analysis demonstrated strong discriminative ability for several physical therapy outcome measures in differentiating fallers from non-fallers among older adults with KOA, with AUC values ranging from 0.78 to 0.86 (Figure 3). The TUG–Dual Task showed the highest overall diagnostic performance (AUC = 0.86, 95% CI: 0.79–0.93), with an optimal cut-off of ≥15.6 s yielding 85.71% sensitivity and 76.19% specificity (*p* < 0.001). The Functional Gait Assessment also performed well (AUC = 0.83, 95% CI: 0.75–0.90), with a threshold of ≤18 points achieving 82.14% sensitivity and 74.60% specificity (*p* < 0.001). The FSST showed good accuracy (AUC = 0.81), while the FES-I demonstrated slightly lower but still significant discriminatory ability (AUC = 0.78), with a cut-off of ≥29 points providing 78.12% sensitivity and 70.24% specificity (*p* = 0.002).

**FIGURE 4:**
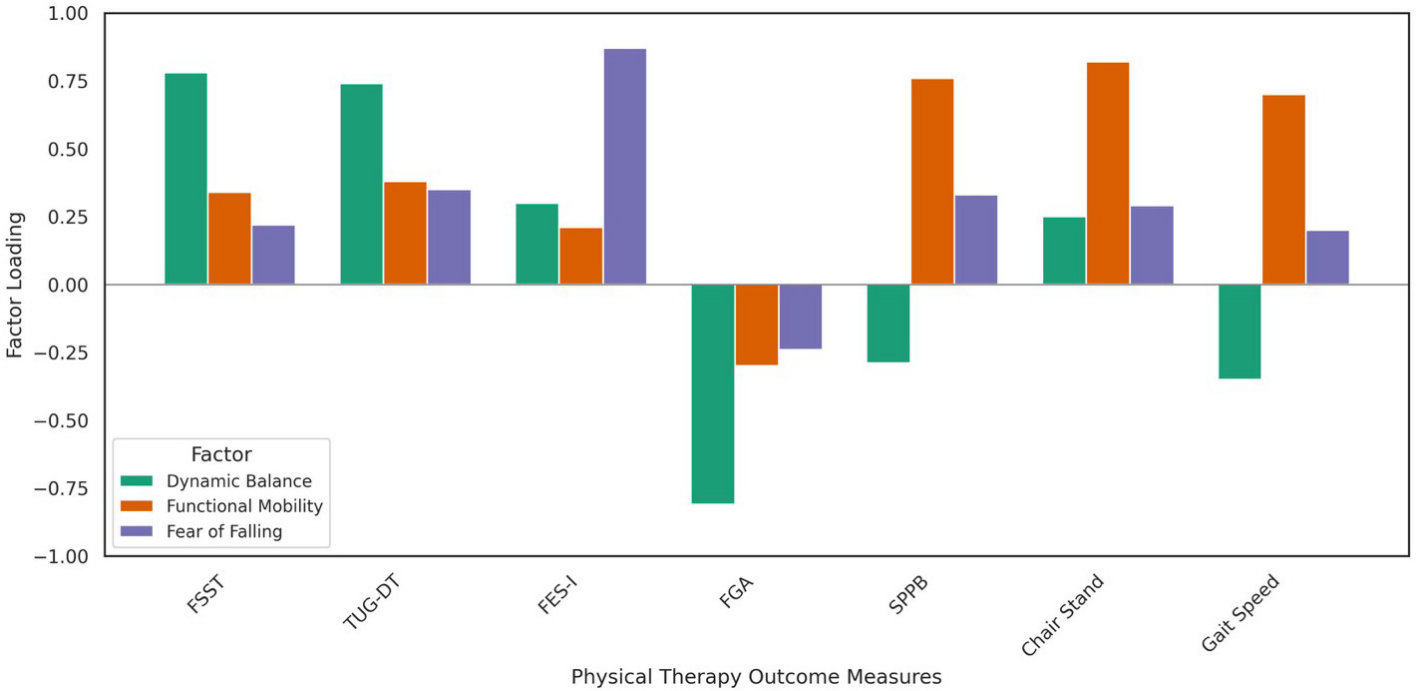
Factor loadings from exploratory factor analysis of physical therapy outcome measures contributing to the neuromotor frailty phenotype in older adults with knee osteoarthritis.

Exploratory factor analysis using principal component analysis with Varimax rotation identified three distinct components underlying the neuromotor frailty phenotype in older adults with KOA, accounting for clinically interpretable functional domains (Figure 4 and Table 4). Factor 1 (“Dynamic Balance”) was primarily defined by strong positive loadings from FSST (0.78) and TUG–Dual Task (0.74), and a strong negative loading from FGA (−0.81), indicating a coherent balance-related construct. Factor 2 (“Functional Mobility”) captured traditional mobility and lower-extremity strength measures, with high loadings from the 30-s chair stand test (0.82), the SPPB (0.76), and gait speed (0.70). Factor 3 (“Fear of Falling”) was uniquely represented by the FES-I, which loaded strongly at 0.87, distinguishing psychological risk perception from physical function.

**TABLE 4:** Exploratory factor analysis (principal component analysis with varimax rotation) of physical therapy outcome measures contributing to the neuromotor frailty phenotype in older adults with knee osteoarthritis.

Physical therapy outcome measure	Factor 1: dynamic balance	Factor 2: functional mobility	Factor 3: fear of falling
Four Square Step Test (FSST)	0.78	0.34	0.22
TUG – Dual Task (TUG-DT)	0.74	0.38	0.35
Falls Efficacy Scale–International (FES-I)	0.3	0.21	0.87
Functional Gait Assessment (FGA)	−0.81	−0.3	−0.24
Short Physical Performance Battery (SPPB)	−0.29	0.76	0.33
30-Second Chair Stand Test	0.25	0.82	0.29
10-Meter Walk Test (Gait Speed)	−0.35	0.7	0.2

EFA, exploratory factor analysis; FSST, Four Square Step Test; FGA, Functional Gait Assessment; SPPB, Short Physical Performance Battery; FES-I, Falls Efficacy Scale–International; TUG-DT, Timed Up and Go – Dual Task.

## 4 Discussion

This study aimed to identify effective physical therapy outcome measures that distinguish fall status among older adults with KOA and to explore the underlying structure of these measures in relation to neuromotor frailty. The results showed that individuals with a recent history of falls performed significantly worse across several clinically relevant domains, including strength, gait, balance, and functional mobility. Several outcome measures—such as the Timed Up and Go Dual Task, Four Square Step Test, and Falls Efficacy Scale–International—were independently associated with fall status, as confirmed by logistic regression. Receiver operating characteristic analysis further demonstrated the diagnostic usefulness of these measures, showing high sensitivity and specificity in identifying fallers from non-fallers. Exploratory factor analysis identified three underlying components—dynamic balance, functional mobility, and fear of falling—that represent distinct but related aspects of the neuromotor frailty phenotype. Overall, these findings underscore the importance of combining performance-based and self-reported measures to interpret fall risk as a multidimensional construct of vulnerability in this clinical population.

Fallers demonstrated significantly worse performance in lower limb strength, gait speed, dynamic balance, and dual-task mobility compared to non-fallers. These findings support previous research indicating that impairments in mobility and postural control are key factors contributing to increased fall risk among older adults with musculoskeletal conditions (43). Boekesteijn et al. (44) noted that reduced quadriceps strength and slower gait are significant functional deficits in individuals with KOA, which directly hinder their ability to recover from perturbations. Similarly, Rice et al. (45) noted that deficits in dynamic balance, as measured by tests such as the FSST and TUG-DT, are closely linked to sensorimotor decline and functional instability in this group. The current study also finds that fallers experience greater fear of falling and lower physical activity levels, both of which are associated with adverse outcomes in older adults (46). These results highlight the complex nature of fall risk in KOA, where neuromotor, psychological, and behavioral factors interact to contribute to functional decline.

Logistic regression analysis revealed that higher scores on the TUG-DT, FSST, and FES-I were independently associated with increased odds of falling. In comparison, higher scores on the SPPB and FGA were associated with a protective effect. These findings align with prior studies demonstrating that dual-task interference, dynamic balance performance, and perceived fall risk are strongly associated with fall occurrence in older adults (23, 47, 48). The ROC analysis further confirmed that these measures, particularly the TUG-DT and FGA, offer strong discriminative accuracy, supporting their clinical relevance. The optimal cutoff identified for the TUG–Dual Task (≥15.6 s) is generally consistent with previously reported thresholds for elevated fall risk in older adults, in which dual-task TUG values exceeding approximately 14–16 s have been associated with impaired mobility and increased fall risk. The slightly higher threshold observed in the present cohort may reflect additional mobility constraints imposed by KOA, including pain, joint stiffness, and reduced lower-limb strength. Similarly, the FGA cutoff of ≤18 points aligns with prior studies suggesting that scores below 18–20 indicate increased fall vulnerability in community-dwelling older adults (49). The dual-task paradigm employed in the present study incorporated a cognitive interference task (alternating alphabet recitation), which primarily challenges attentional allocation and executive function during gait (2). Given that knee osteoarthritis is predominantly associated with pain, joint instability, and altered biomechanics, a motor–motor dual-task condition (ambulating while carrying an object) may impose different mechanical and postural control demands and could potentially produce distinct performance patterns. Future research should compare cognitive–motor and motor–motor dual-task paradigms to determine whether task specificity differentially influences mobility performance and fall risk in this population.

### 4.1 Clinical significance

This study demonstrates that a focused set of physical therapy outcome measures—namely the Timed Up and Go–Dual Task (TUG–DT), Four Square Step Test (FSST), Functional Gait Assessment (FGA), Short Physical Performance Battery (SPPB), and Falls Efficacy Scale–International (FES-I) effectively distinguishes fallers from non-fallers among older adults with knee osteoarthritis (KOA). The identification of three distinct domains—dynamic balance, functional mobility, and fear of falling—provides a clear and clinically meaningful framework for characterizing neuromotor frailty in this population. Together, these findings support the routine use of multidimensional assessment strategies in physical therapy practice, enabling clinicians to identify high-risk individuals using standardized, accessible, and scalable tools.

Beyond classification, the strong discriminative performance of these measures informs targeted rehabilitation planning. In routine outpatient musculoskeletal care, older adults with KOA should undergo structured fall-risk screening that includes a dual-task mobility assessment (TUG–DT), a multidirectional stepping evaluation (FSST), a standardized gait assessment (FGA), lower-extremity functional testing (SPPB or 30-s chair stand), and a fear-of-falling evaluation (FES-I). The particularly high diagnostic accuracy of the TUG–DT and FGA supports their use as core components of both initial assessment and follow-up evaluations. Systematic integration of these measures into clinical workflows facilitates early identification of individuals exhibiting a neuromotor frailty phenotype, potentially before recurrent falls occur. Rehabilitation strategies should then be aligned with the three identified domains, ensuring that interventions address not only strength and gait impairments but also dual-task interference and psychological vulnerability. This domain-based approach offers a structured, evidence-informed model for individualized fall-prevention programming in older adults with KOA.

### 4.2 Limitations and areas for future research

Several limitations should be acknowledged when interpreting these findings. First, the sample comprised community-dwelling older adults recruited from a single outpatient clinic in Saudi Arabia, which may limit generalizability to other geographic or cultural contexts. Cultural norms related to physical activity participation, gender roles, and social engagement, as well as environmental factors such as climate and built environment, may influence habitual activity levels as reflected by MET-min/week. Additionally, differences in healthcare access, health education, and fall-prevention services may shape perceptions of fall risk and fear of falling as measured by the FES-I. Therefore, caution is warranted when extrapolating these findings to populations in other regions with differing sociocultural and healthcare system characteristics. The cross-sectional design precludes establishing causality between impairments and fall risk, and reliance on retrospective fall histories may be affected by recall bias. Although a calendar landmarking technique was used to enhance memory retrieval, underreporting or inaccurate recall of fall events remains possible, particularly for non-injurious or less salient falls. Such misclassification may have influenced group allocation and attenuated the observed associations between physical therapy outcomes and fall status. Future studies should consider prospective fall monitoring using fall diaries, monthly follow-up contacts, or wearable sensor-based detection systems to improve classification accuracy and strengthen internal validity. The sample consisted of community-dwelling older adults with KOA, which may limit the generalizability of the results to other clinical populations or care settings, such as long-term care or post-acute rehab. Furthermore, individuals with cognitive impairment and neurological or vestibular disorders were excluded to ensure safe participation and a valid performance-based assessment. While methodologically justified, this exclusion limits the generalizability of the findings to broader high-risk fall populations, as many older adults at greatest risk of falls present with cognitive decline, executive dysfunction, stroke, Parkinsonian syndromes, or other neurological conditions that substantially influence balance and dual-task performance. Consequently, the neuromotor frailty phenotype identified in this study should be interpreted as applicable primarily to cognitively intact older adults with KOA. Future research should examine whether similar factor structures and associations are observed in more clinically complex populations. Additionally, although the study employed a comprehensive set of outcome measures, several relevant domains—such as vestibular function, proprioception, and cognitive flexibility—were not assessed. Future research should employ longitudinal designs to assess better how well these measures predict outcomes over time and should consider using sensor-based technologies to improve measurement accuracy. Exploring how interventions respond within each identified factor domain could also enhance rehabilitation strategies and outcome monitoring.

## 5 Conclusion

This study shows that specific physical therapy outcome measures are clinically meaningful and statistically robust in differentiating fallers from non-fallers and in characterizing neuromotor frailty in older adults with KOA. The use of performance-based and self-report assessments together enabled the identification of three distinct functional domains, each of which independently contributes to fall vulnerability. These findings support the routine use of standardized outcome measures in geriatric musculoskeletal practice to help with early detection, risk stratification, and management of fall risk in this high-risk group.

## Data Availability

The datasets presented in this study can be found in online repositories. The names of the repository/repositories and accession number(s) can be found in the article/Supplementary material.
